# The impact of vancomycin susceptibility on treatment outcomes among patients with methicillin resistant *Staphylococcus aureus *bacteremia

**DOI:** 10.1186/1471-2334-11-335

**Published:** 2011-12-05

**Authors:** Hitoshi Honda, Christopher D Doern, Wm Michael-Dunne, David K Warren

**Affiliations:** 1 Division of Infectious Diseases, Department of Medicine, Washington University School of Medicine, St. Louis, Missouri, USA; 2 Department of Pathology, University of Texas Southwestern Medical Center, Dallas, Texas, USA; 3 Department of Pathology and Immunology, Washington University School of Medicine, St. Louis, Missouri, USA

## Abstract

**Background:**

Management of methicillin-resistant *Staphylococcus aureus *(MRSA) bacteremia remains a challenge. The emergence of MRSA strains with reduced vancomycin susceptibility complicates treatment.

**Methods:**

A prospective cohort study (2005-2007) of patients with MRSA bacteremia treated with vancomycin was performed at an academic hospital. Vancomycin minimum inhibitory concentration (MIC) and minimum bactericidal concentration (MBC) were determined for stored MRSA isolates. Cox regression analysis was performed to predict 28-day all-cause mortality.

**Results:**

One hundred sixty-three patients with MRSA bacteremia were evaluated. One hundred twelve patients (68.7%) had bacteremia due to MRSA with a vancomycin MIC ≥ 2 *ug*/mL. Among strains with a vancomycin MIC ≥ 2 *ug*/mL, 10 isolates (8.9%) were vancomycin-intermediate *S. aureus *(VISA). Thirty-five patients (21.5%) died within 28 days after the diagnosis of MRSA bacteremia. Higher vancomycin MIC was not associated with mortality in this cohort [adjusted hazard ratio (aHR), 1.57; 95% confidence interval (CI), 0.73-3.37]. Vancomycin tolerance was observed in 4.3% (7/162) of isolates and was not associated with mortality (crude HR, 0.62; 95% CI, 0.08-4.50). Factors independently associated with mortality included higher age (aHR, 1.03; 95% CI 1.00-1.05), cirrhosis (aHR, 3.01; 95% CI, 1.24-7.30), and intensive care unit admission within 48 hours after the diagnosis of bacteremia (aHR, 5.99; 95% CI, 2.86-12.58).

**Conclusions:**

Among patients with MRSA bacteremia treated with vancomycin, reduced vancomycin susceptibility and vancomycin tolerance were not associated with mortality after adjusting for patient factors. Patient factors including severity of illness and underlying co-morbidities were associated with the mortality.

## Background

Methicillin-resistant *Staphylococcus aureus *(MRSA) bacteremia is a common infection. In a recent epidemiological study of nine US communities, the pooled mean incidence of MRSA bacteremia was 2.24 per 10, 000 population per year [1]. MRSA bacteremia is increasingly seen in the general community [2]. MRSA bacteremia is often complicated by medical device involvement, metastatic infection, recurrence, and mortality. MRSA bacteremia is also associated with a worse outcome compared to bacteremia caused by methicillin-susceptible *S. aureus *[3-5]. Management of MRSA bacteremia is a challenge since clinical cure may not be achieved in selected cases, despite the appropriate antimicrobial therapy [6]. Vancomycin remains the mainstay treatment for MRSA bacteremia [7]. However, reduced vancomycin susceptibility may be associated with worse clinical outcomes (i.e., mortality or septic shock) among patients with MRSA bacteremia [8-10]. This observation led in part to the lowering of the breakpoint for vancomycin susceptibility to ≤ 2 *μg*/mL by the Clinical and Laboratory Standards Institute (CLSI) in 2006 [11]. However, recent studies have demonstrated a variable association between vancomycin MIC and clinical outcome [12-14]. Some of this variability may be explained by other phenotypic characteristics such as vancomycin minimal bactericidal concentration (MBC) or vancomycin tolerance [15,16]. The purpose of our study is to determine the impact of phenotypic characteristics on mortality of MRSA bacteremia using comprehensive data (2005-2007) from large prospective cohort at US tertiary hospital.

## Methods

A prospective cohort study of patients with MRSA bacteremia was conducted between July 2005 and July 2007 at Barnes-Jewish Hospital, a 1252-bed, academic, tertiary care centre in St. Louis, Missouri. Diagnosis of MRSA bacteremia was defined as ≥ 1 blood culture positive for MRSA. Patients who did not receive vancomycin as initial therapy or were less than 18 years old at the time of diagnosis of MRSA bacteremia were excluded. In order to analyze incident cases of invasive MRSA infections, patient who had a history of MRSA bacteremia in the previous three months, or a history of MRSA endocarditis in the twelve months prior to initial positive blood culture at our hospital during the study period were also excluded. Isolates from MRSA bacteremia were individually labeled and separately stored at - 40° C for microbiological analysis.

### Microbiological analysis

Minimum inhibitory concentrations (MIC) were determined by microbroth dilution method, using the SIEMENS (West Sacramento, CA) MicroScan^® ^- Pos MIC Panel Type 26 as a testing platform. Briefly, MicroScan^® ^panels were inoculated according to manufacturer's instruction using colonies of *Staphylococcus aureus *grown overnight on Remel tryptic soy agar blood agar plates (Lenexa, KS). Panels were incubated at 35°C in room air. Panels were run in full manual mode, and all microbroth dilution panels were manually read by a single investigator (C.D.C.) and MIC data was recorded for each isolate at 24 hours. The vancomycin doubling dilution range for this assay was from 0.25 to 16 mg/L. *Enterococcus faecalis *ATCC 29212 and *Staphylococcus aureus *ATCC 29213 strains were used for quality control using the reference ranges provided in the package insert.

The MBC values were obtained using the method of Sader, et al. [17] The minimum bactericidal concentrations (MBC) for vancomycin were defined as 99.9% killing of the initial inocula after 24 hours of incubation. Following 24 hours of incubation, colony counts were conducted from the MIC well and every well thereafter demonstrating no bacterial growth. After thoroughly re-suspending each well, two aliquots of 20 μl were plated to TSA blood agar plates for isolation and subsequent colony counts. An average of the two aliquots was multiplied by the dilution factor (1:5). The MBC was established as the first well with less than 99.9% of the initial inoculum. Repeat testing was conducted on those isolates with MBCs > 16 *μg*/mL to confirm the result. Vancomycin- intermediate *S. aureus *was defined as strains having a vancomycin MIC of 4 or 8 *μg*/mL by microbroth dilution testing [11]. Vancomycin tolerance was defined by either an MBC: MIC ratio ≥ 32 or an MBC: MIC ratio of 16 associated with a resistant-level vancomycin MBC (≥ 32 *μg*/mL) [16-18].

### Definitions and data collection

Patients were identified by daily review of microbiology reports. Demographic characteristics and clinical data were prospectively obtained from the medical record. A written informed consent from the patients was waived for this observational study. We tracked all-cause mortality for 28 days after the first blood culture positive for MRSA by reviewing both medical records and the Social Security Death Index [19]. If the patient did not die on initial hospital stay, then any patients readmitted > 28 days after the initial diagnosis of MRSA bacteremia were considered to be alive at 28 days. If there were no readmission data > 28 days after diagnosis of MRSA bacteremia available, Social Security Death Index was used to determine if patients died. Metastatic infection was defined by microbiological or radiographic evidence of remote site of infection consistent with hematogenous spread [20]. Healthcare-associated, hospital-onset bacteremia was defined by a positive blood culture obtained from patients who were hospitalized > 48 hours [21]. Healthcare-associated, community onset of bacteremia was defined as a positive blood culture ≤ 48 hours after hospitalization with the following criteria; presence of an invasive device, history of MRSA infection or colonization, surgery, hospitalization, dialysis, or residence in a long-term care facility in the 12 months preceding the culture [21]. Community-associated bacteremia was defined by a positive blood culture ≤ 48 hours without meeting the criteria of healthcare associated community-onset bacteremia [21].

### Statistical analyses

Vancomycin MIC was dichotomized (MIC of ≤ 1 *μg*/mL and MIC ≥ 2 *μg*/mL). Comparison between categorical variables was done using Chi-square or Fisher's exact test. Continuous variables were compared using the Mann-Whitney test. Two-tailed Spearman's rho was used to calculate correlation coefficients between vancomycin MIC and MBC. All tests for significance were 2-tailed, with *P *values < 0.05 considered significant.

We performed multivariable survival analysis to predict 28-day all-cause mortality. Potential risk factors for 28-day all-cause mortality were first assessed by bivariate analysis. A Cox proportional hazards model was developed in forward stepwise fashion to predict mortality. The variable of vancomycin MIC (either ≤ 1 *μg*/mL or ≥ 2 *μg*/mL) was forced into the final model since this was the independent variable of interest. Variables with *P *< 0.10 in bivariate analysis were considered for inclusion in the models. The assumption of proportional hazards was confirmed by log-log survival plots for independent variables in the final model. A first-order interaction term of initial vancomycin trough level (< or ≥ 15 *μg/*dL) and vancomycin MIC was created to determine if a lower vancomycin trough and higher vancomycin MIC independently increased mortality. Other variables were retained in the final model if *P *< 0.05. All analyses were performed using SPSS version 17.0 (SPSS Inc, Chicago, IL). The Washington University Human Research Protection (institutional review board) approved this project.

## Results

One hundred-sixty three patients met study criteria during the 25-month study period and were included in the analysis. The vancomycin MIC was ≤ 1 *μg*/mL for 51 (31.3%) isolates, and ≥ 2 *μg*/mL for 112 (68.7%) isolates (MIC_50 _= 2 *μg*/mL). Ten isolates (6.1%) had a vancomycin MIC of 4 *μg*/mL. Only one isolate had a vancomycin MIC of 0.5 *μg*/mL. There were no isolates with a vancomycin MIC > 4 *μg*/mL in this cohort.

The vancomycin MBC for all isolates ranged from 1 to ≥ 32 *μg*/mL (MBC_50 _and MBC_90 _were 2 *μg*/mL and 8 *μg*/mL, respectively, Table 1). Seven (4.3%) isolates met the criteria for vancomycin tolerance, while the MBC: MIC ratio to determine vancomycin tolerance for one isolate could not be determined (The strain with a vancomycin MIC of 4 *μg*/mL and MBC ≥ 32 *μg*/mL). Overall, vancomycin MIC was highly correlated with vancomycin MBC (r = 0.734, p < 0.001). A comparison of study patients by vancomycin MIC is shown in Table 2. There were no significant differences in patient characteristics between patients infected with MRSA isolates having low versus high vancomycin MICs.

**Table 1:** Vancomycin Minimum Inhibitory Concentration and Minimum Bactericidal Concentration for 163 Isolates of Methicillin-Resistant *S. aureus *Bacteremia

		Vancomycin MBC (*ug*/mL)					
**Vancomycin MIC** **(*ug*/mL)**	**No. of isolates (%)** **(n = 163)**	**1**	**2**	**4**	**8**	**16**	**≥32**

< 1	1 (0.6)	----	1	----	----	----	----
1	50 (30.6)	37	12	----	----	1	----
2	102 (62.6)	----	75	14	6	----	7^a^
4	10 (6.1)	----	----	7	1	1	1^b^

MIC, minimum inhibitory concentration; MBC, minimum bactericidal concentration.

a. Seven isolates were met the criteria for vancomycin tolerance.

b. MBC: MIC ratio for one isolate was undetermined.

**Table 2:** Comparison of 163 Patients with Methicillin-Resistant *Staphylococcus aureus *Bacteremia, by Vancomycin Minimum Inhibitory Concentration.

Variable	MIC (*ug*/mL)		
	≤ 1 (n = 51)	≥ 2 (n = 112)	*p*
Age, years, median (range)	54 (26-95)	59 (23-92)	.115
Female gender	23 (45.1)	60 (53.6)	.316
White race	22 (43.1)	51 (45.5)	.775
Congestive heart failure	10 (19.6)	25 (22.3)	.696
Coronary artery disease	11 (21.6)	26 (23.2)	.817
Chronic obstructive pulmonary diseases	15 (29.4)	22 (19.6)	.167
Renal function			
Normal	36 (70.6)	81 (72.3)	Ref.^c^
Chronic renal insufficiency without dialysis	4 (7.8)	10 (8.9)	.866
Chronic renal insufficiency with dialysis	11 (21.6)	21 (18.8)	.697
Malignancy	10 (19.6)	25 (22.3)	.696
HIV infection	4 (7.8)	3 (2.7)	.206
Chronic skin disease	5 (9.8)	8 (7.1)	.547
Peripheral vascular diseases	4 (7.8)	7 (6.3)	.741
Diabetes mellitus	27 (52.9)	34 (30.4)	.006
Systemic corticosteroid use last 28 days	9 (17.6)	15 (13.4)	.477
Cirrhosis	4 (7.8)	10 (8.9)	1.00
History of intravenous drug use	4 (7.8)	11 (9.8)	.778
History of smoking	29 (56.9)	67 (58.9)	.865
Any transplant	2 (3.9)	8 (7.1)	.726
Surgery during hospitalization	10 (19.6)	13 (11.6)	.174
Prosthetic joint	4 (7.8)	11 (9.8)	.778
Vascular graft	9 (17.6)	14 (12.5)	.381
Cardiac device	4 (7.8)	6 (5.4)	.506
ICU admission within 48 hours after the first positive blood culture	19 (37.3)	40 (35.7)	.849
Central venous catheterization at the time of the first (+) blood culture	37 (72.5)	77 (68.8)	.624
Onset			
Community-associated	4 (7.8)	8 (7.1)	Ref.^c^
Healthcare-associated community onset	32 (62.7)	66 (58.9)	.962
Healthcare-associated hospital onset	15 (29.4)	38 (33.9)	.730
Site of MRSA bacteremia			
Primary bacteremia	25 (49.0)	45 (40.2)	Ref^c^
Catheter related bloodstream infection	12 (23.5)	25 (22.3)	.344
Skin infection	8 (15.7)	17 (15.2)	.508
Postoperative surgical site infection	2 (3.9)	12 (10.7)	.552
Other	4 (7.8)	13 (11.6)	.520
Any metastatic infection at the time of diagnosis	6 (11.8)	17 (15.2)	.562
Outcome			
Recurrent MRSA infection (n = 161) ^a, b^	10/51 (19.6)	16/110 (14.5)	.417
Death at 28 days after diagnosis of bacteremia	9 (17.6)	26 (23.2)	.422

HIV, human immunodeficiency virus; ICU, intensive care unit; MRSA, methicillin-resistant *S. aureus*.

Ref, reference; MIC, minimum inhibitory concentration.

a. See methods for definition.

b. Two patients were excluded from analysis since they died within 48 hours of diagnosis.

c. *P *value determined using univariate logistic regression.

Percentage of each column with variable is shown in parentheses, unless otherwise stated.

The 28-day all-cause mortality rate for the cohort was 21.5%. Median length of time from the time of first positive blood culture to death was 13 days (range; 1-28 days). Predictors of 28-day mortality of MRSA bacteremia are shown in Table 3. Increased age, chronic renal insufficiency without dialysis, cirrhosis, vascular graft, central venous catheter at the time of bacteremia, ICU admission within 48 hours at the time of first positive blood culture, and vancomycin MIC > 2 *μg*/mL were considered for inclusion in the multivariate model. In the multivariate model, factors independently associated with mortality were increased age [adjusted hazard ratio (aHR), 1.03 per year; 95% confidence interval (CI), 1.00-1.05], cirrhosis (aHR, 3.01; 95% CI, 1.24-7.30), and ICU admission within 48 hours of diagnosis (aHR, 5.99; 95% CI, 2.86-12.58). Lower initial vancomycin trough level (i.e., < 15 *μg/*dL) was not associated with 28-day mortality (n = 151, crude HR, 0.77; 95% CI, 0.36-1.63). MRSA phenotype characteristics, both vancomycin susceptibility ≥ 2 *μg*/mL (adjusted HR, 1.57; 95% CI, 0.73-3.37) and vancomycin tolerance (n = 162, crude HR, 0.62; 95% CI, 0.08-4.50) were not associated with 28-day mortality.

**Table 3:** Predictors of 28-day All-Cause Mortality after Diagnosis of Methicillin-Resistant *Staphylococcus aureus *Bacteremia for 163 Patients

Variable	Death (n = 35)	Alive (n = 128)	Crude HR (95% CI)	Adjusted HR (95% CI)
Age, years, median (range)	64 (33-92)	56 (23-95)	1.03 (1.00-1.05)	1.03 (1.00-1.05)
Female gender	16 (45.7)	67 (52.3)	0.83 (0.43-1.61)	
White race	20 (57.1)	70 (54.7)	1.05 (0.54-2.05)	
Congestive heart failure	9 (25.7)	26 (20.3)	1.33 (0.62-2.84)	
Coronary artery disease	8 (22.9)	29 (22.7)	0.99 (0.45-2.19)	
Chronic obstructive pulmonary diseases	10 (28.6)	27 (21.1)	1.43 (0.69-2.97)	
Renal function				
Normal	25 (71.4)	92 (71.9)	Reference	
CRI without dialysis	7 (20.0)	7 (5.5)	2.67 (1.15-6.17)	
CRI with dialysis	3 (8.6)	29 (22.7)	0.42 (0.13-1.37)	
Malignancy	8 (22.9)	27 (21.1)	1.08 (0.49-2.37)	
HIV infection	1 (2.9)	6 (4.7)	0.58 (0.08-4.26)	
Chronic skin disease	2 (5.7)	11 (8.6)	0.69 (0.17-2.87)	
Peripheral vascular diseases	2 (5.7)	9 (7.0)	0.81 (0.19-3.37)	
Diabetes mellitus	10 (28.6)	51 (39.8)	0.64 (0.31-1.32)	
Systemic corticosteroid use last 28 days	8 (22.9)	16 (12.5)	2.01 (0.91-4.42)	
Cirrhosis	6 (17.1)	8 (6.3)	2.50 (1.04-6.02)	3.01 (1.24-7.30)
History of intravenous drug use	2 (5.7)	13 (10.2)	0.57 (0.14-2.37)	
History of smoking	22 (62.9)	73 (57.0)	1.27 (0.64-2.52)	
Alcohol use	11 (31.4)	39 (30.5)	1.06 (0.52-2.15)	
Any transplant	2 (5.7)	8 (6.3)	0.89 (0.22-3.73)	
Surgery during hospitalization	4 (11.4)	19 (14.8)	0.78 (0.28-2.21)	
Prosthetic joint	0	15 (11.7)	0.04 (0-5.06)	
Vascular graft	1 (2.9)	22 (17.2)	0.16 (0.02-1.16)	
Cardiac device	3 (8.6)	7 (5.5)	1.40 (0.43-4.56)	
ICU admission within 48 hours after the first positive blood culture	25 (71.4)	34 (26.6)	5.56 (2.67-11.60)	5.99 (2.86-12.58)
Central venous catheterization at the time of the first positive blood culture	29 (82.9)	85 (66.4)	2.25 (0.93-5.42)	
Onset				
Community-associated	3 (8.6)	9 (7.0)	Reference	
Healthcare-associated community onset	21 (60.0)	77 (60.2)	0.76 (0.23-2.55)	
Healthcare-associated hospital onset	11 (31.4)	42 (32.8)	0.75 (0.21-2.67)	
Any metastatic infection at the time of diagnosis	6 (17.1)	17 (13.3)	1.27 (0.53-3.05)	
Vancomycin tolerance (n = 162) ^a, b^	1/35 (2.9)	6/127 (4.7)	0.62 (0.08-4.50)	
Vancomycin initial trough < 15 *mg*/L (n = 151)^c^	14/27 (51.9)	73/124 (58.9)	0.77 (0.36-1.63)	
Vancomycin MIC ≥ 2 *ug*/mL	26 (74.3)	86 (67.2)	1.36 (0.64-2.89)	1.57 (0.73-3.37)

CRI, chronic renal insufficiency; HIV, human immunodeficiency virus; ICU, intensive care unit;

MRSA, methicillin-resistant *S. aureus*; HR, hazard ratio; MIC, minimum inhibitory concentration.

Variables considered but not retained in the final model were renal function (chronic renal failure without dialysis), vascular graft, central venous catheterization at the time of diagnosis, and vancomycin MIC ≥2 *ug*/mL.

a. See methods for definition.

b. One patient was excluded since MBC: MIC ratio was undetermined.

c. Twelve patients did not have an initial vancomycin trough level. [unknown reason (n = 7) or died before initial trough was obtained (n = 5)]

## Discussion

In contrast to previous studies, [8-10] we did not observe an association between 28-day all-cause mortality and increased vancomycin MIC (≥ 2 *μg*/ml) or vancomycin tolerance among patients with MRSA bacteremia, after adjusting for underlying patient factors.

In this prospective cohort, the incidence of MRSA bacteremia due to strains with a vancomycin MIC of 2 *μg*/ml and VISA bacteremia (i.e., MIC ranging 4-8 *μg*/ml) was 62.6% and 6.1%, respectively. According to 2005 Surveillance Network data from a large sample of US laboratories, *S. aureus *isolates with reduced susceptibility to vancomycin was rare; the percentage of over 240, 000 isolates with vancomycin MIC of 2 *μg*/ml and vancomycin MIC ≥ 4 was 16.2% and 0.2%, respectively [11]. Musta et al. showed in their cohort, the percentage of MRSA blood culture isolates with a vancomycin MIC of 2 *μg*/ml (including 1.5 *μg*/ml) and > 2 *μg*/ml was 82.8% and 2.7%, respectively [13]. The reason for our cohort having a higher prevalence of strains with reduced vancomycin susceptibility and VISA was not completely clear. The current prevalence of VISA in the US is unknown. Since our cohort included the data from 2007, this might be indicative of an overall increase in vancomycin MIC among MRSA. However, a large sample of isolates must be tested to confirm this.

In previous studies, Soriano et al, demonstrated progressively higher vancomycin MICs (ranging between 1, 1.5, and 2 *μg*/ml) were associated with increased 30-day mortality when vancomycin was used to treat MRSA bacteremia [10]. A paradoxical relationship (i.e., increased vancomycin MIC was correlated with better clinical outcome) was also observed in one study [14]. The discordant finding of these various studies has generated several hypotheses, including a potential difference in strain virulence due to geographic location. Although a trend towards increased mortality was observed among our few patients infected with MRSA isolates with vancomycin MIC of 4 *μg*/ml, this was not statistically significant (data not shown).

Among the 151 patients in which an initial vancomycin trough was obtained, a lower vancomycin trough level was not associated with increased mortality in our cohort, which has been reported by others [22]. Treatment failure of MRSA bacteremia using vancomycin raises questions about the ideal serum vancomycin concentration for treatment of *S. aureus *bacteremia. Based on limited animal studies and Monte Carlo simulation studies, the targeted therapeutic range of vancomycin [area under the concentration-versus-time curve (AUC): MIC of ≥ 400] is not achievable with conventional vancomycin dosing if the MIC is ≥ 2 *μg*/ml in patients with normal renal function [23,24]. This led to a recommendation not to use vancomycin for a AUC:MIC ratio ≥ 400. However, there is no definitive human clinical data to support this recommendation [24]. Moreover, nephrotoxicity is a concern when a higher dose of vancomycin is administered [25]. We created a model with an interaction variable for both higher vancomycin (≥ 2 *μg*/ml) and lower initial vancomycin trough level (< 15 mg/L), however this interaction variable was not associated with increased mortality (data not shown). Our finding supports the hypothesis that vancomycin MIC of 2 *μg*/ml may not always predict worse outcome in patients with MRSA bacteremia.

The effect of vancomycin MBC and vancomycin tolerance on patient outcome have rarely been examined in clinical practice. Vancomycin tolerance may be associated with reduced efficacy of antimicrobials (i.e., bactericidal or bacteriostatic activity) based on animal models [26]. In our cohort, the incidence of vancomycin tolerance was approximately 4%. We did not observe an association between vancomycin tolerance and increased mortality although we had small numbers of vancomycin-tolerant isolates in our cohort. In our study, vancomycin MBC was highly correlated with vancomycin MIC; over 70% of isolates revealed an identical value of vancomycin MIC and MBC. Based on our findings, MBC and vancomycin tolerance is likely to be of limited value as a predictor of outcomes in MRSA bacteremia patients treated with vancomycin.

Our study demonstrated that patient factors, such as patient's age, co-morbidities, presence of devices, and ICU admission ≤ 48 hours after first positive blood culture are predictive of short-term mortality associated with MRSA bacteremia, similar to what has been noted by others [27-29]. This suggests that earlier identification of high risk patients, and timely management in the early stage of illness are important in reducing mortality in MRSA bacteremia.

Our study has several limitations. It occurred in a single, tertiary care center, and may not be generalizable to other hospitals. The extent of diseases associated with MRSA bacteremia was not fully evaluated in this study, especially the presence of endocarditis, since not all patients with MRSA bacteremia underwent further evaluation such as transesophageal echocardiogram [30]. In this cohort, transesophageal echocardiogram was only performed for only 17.2% (18/163) of patients. Clinical information at the time of diagnosis, such as, intensive care unit admission 48 hours or less at the time of diagnosis, active malignancy, and cirrhosis was used as a predictor of the severity of the illness. We were unable to collect information to calculate Acute Physiology and Chronic Health Evaluation II score and Charlson comorbidity index which may be more appropriate for a predictor of the severity of illness. Although vancomycin trough level was are generally obtained before the fourth dose at our institution, 7.4% of patients in our study did not have a trough level drawn, and the timing of when vancomycin trough level were obtained for the remainder of other cohort was not standardized. Persistent bacteremia (i.e., > 7 days bacteremia) [31] occurred in only one patient in our cohort; therefore, we were unable to assess the relationship between vancomycin MIC and duration of bacteremia. Despite having prospective data on over 163 patients and an overall mortality rate of 21.5%, our study is not powered to detect small differences in mortality due to infection from MRSA isolates with either low or high vancomycin MICs. While our data did not support a direct relationship vancomycin MIC and mortality in patients with MRSA bacteremia, this finding may not apply to other invasive MRSA infection, especially those involving other organs (e.g., central nervous system, bone, and eye) in which the physiologically achievable vancomycin concentration is lower than in blood. Due to the low incidence of VISA bacteremia in our cohort, we were unable to assess the impact of VISA on mortality.

## Conclusion

We found that reduced vancomycin susceptibility and vancomycin tolerance were not associated with significant differences in 28-day mortality among a cohort of patients with MRSA bacteremia. Other patient risk factors are likely associated with unfavorable clinical outcome. Even with the rising incidence of infections due to MRSA strains with reduced vancomycin MIC, vancomycin is still the first-line agent for treating MRSA bacteremia. Our data suggest that for bacteremia due to MRSA with a vancomycin MIC of 2 *μg*/mL, vancomycin may still remain a viable treatment option.

## Competing interests

DKW reports that he is a consultant for 3M Healthcare and Cardinal Health and receives research funding from Sage Products, 3M Healthcare, bioMeriéux, and Cubist Pharmaceuticals

All other authors report no conflicts of interest relevant to this article.

## Authors' contributions

HH collected the clinical information of cohort data and participated in the design of the study, performed statistical analysis, and prepared manuscript.

CDD performed phenotypic testing of isolates.

WMD participated in the design of the study and performed phenotypic testing of isolates.

DKW conceived of the study, and participated in its design and coordination and helped to draft the manuscript. All authors read and approved the final manuscript.

## Pre-publication history

The pre-publication history for this paper can be accessed here:

http://www.biomedcentral.com/1471-2334/11/335/prepub
